# The Origin of Cancer-Associated Fibroblasts (CAFs) in Brain Metastases: Seven Hypotheses and Current Evidence

**DOI:** 10.3390/cancers18152397

**Published:** 2026-07-25

**Authors:** Dave Bandke, Rupert Langer

**Affiliations:** 1 Department of Pathology and Molecular Pathology, Johannes Kepler University Linz, Kepler University Hospital GmbH, Wagner-Jauregg-Weg 15, 4020 Linz, Austria; rupert.langer@jku.at; 2 Clinical Research Institute for Neurosciences, Johannes Kepler University, Neuromed Campus, 4020 Linz, Austria

**Keywords:** brain metastasis, cancer-associated fibroblasts, CAF-like cells, desmoplastic stroma, tumor microenvironment, stroma

## Abstract

Brain metastases have traditionally been investigated primarily with regard to interactions between tumor cells, the surrounding brain microenvironment, and associated inflammatory responses. By contrast, the presence of a true intratumoral stromal compartment in brain metastases has received much less attention, partly because the normal brain parenchyma lacks classical interstitial fibroblasts. However, recent histological, transcriptomic, and spatial studies suggest that at least a subset of carcinoma brain metastases contains fibroblast-like or cancer-associated fibroblast-like cells associated with collagen-rich extracellular matrix deposition. This raises a fundamental biological question: where do these cells come from? In this review, we discuss seven non-mutually exclusive hypotheses for the origin of cancer-associated fibroblast (CAF)-like cells in brain metastases, including pseudostromal mimics, meningeal or perivascular fibroblast-related cells, vascular mural cells, glial cells, cancer stem cell plasticity, tumor–stroma clusters, and circulating mesenchymal precursors. We compare the current findings for each hypothesis and highlight their major limitations. Overall, the available data support a heterogeneous and context-dependent model rather than a single universal origin. A better understanding of these stromal cell states may help refine the biological classification of brain metastases and support the future development of more targeted stromal therapies.

## Simple Summary

Research in brain metastasis has long focused on how tumor cells interact with brain cells and the immune system, while much less attention has been paid to the supportive connective tissue. However, some carcinoma metastases build such a stroma within the tumor. This is surprising, because the normal brain, unlike most other organs, does not contain the typical connective tissue with its fibroblasts and adipocytes. Yet a substantial proportion of brain metastases still develop fibroblast-like cells, known as cancer-associated fibroblasts (CAFs), that produce a dense, collagen-rich stroma. This raises a basic question: where do these cells come from? In this review, we gather the current evidence and describe seven possible origins. Understanding these origins may help classify brain metastases more precisely and guide future treatments aimed at the tumor’s supportive environment.

## 1. Introduction

Brain metastases (BMs) have traditionally been studied mainly in terms of tumor–brain interactions, with particular attention to reactive astroglia, microglia, and the invasive front. In this framework, the term “stroma” has rarely been applied in a strict histopathological sense and, when used, has often referred to the surrounding reactive brain tissue rather than to a distinct intratumoral stromal compartment [1].

This conceptual ambiguity is particularly relevant in the brain, because normal parenchyma lacks classical interstitial fibroblasts found in most extracranial organs. Accordingly, the presence of collagen-rich, fibroblast-like, or CAF-like stromal areas in BMs cannot be interpreted simply as a nonspecific result of local tissue remodeling but instead raises the more fundamental question of how such stromal compartments arise in an organ without fibroblasts in the conventional histological sense.

Historically, neuropathologists recognized that BMs often differ from primary glial neoplasms by forming relatively well-circumscribed lesions surrounded by a reactive “glial pseudocapsule” [2]. Immunohistochemical studies in the 1980s first demonstrated collagen deposition, including collagen IV, at the tumor–brain interface [3,4]. Furthermore, original articles in the early 2000s showed that BMs have different infiltration patterns and are not always microscopically clearly demarcated with a gliotic capsule [5,6].

**Figure 1:**
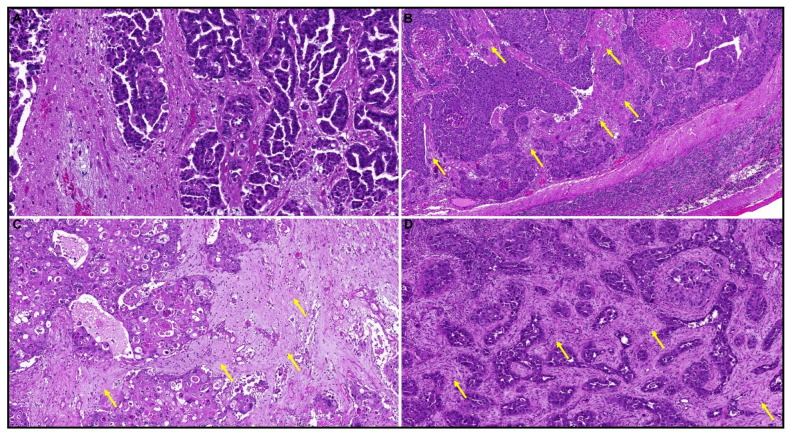
(**A**–**D**) Representative hematoxylin and eosin (H&E)-stained images (10× objective) illustrating the morphological spectrum of intratumoral stroma in BM from our archive: (**A**) Breast carcinoma metastasis without intratumoral stroma, showing the interface with the frontal lobe parenchyma and reactive astrocytes. (**B**) Sharply demarcated breast carcinoma metastasis in the cerebellar white matter with stroma (yellow arrows) confined to the metastatic lesion. (**C**) Deep parenchymal metastasis of pulmonary squamous cell carcinoma with extensive grayish, desmoplastic, relatively paucicellular, partly edematous stroma (yellow arrows). (**D**) Pulmonary squamous cell carcinoma metastasis with meningeal contact and extensive grayish desmoplastic stroma (yellow arrows) of higher cellularity.

Current evidence indicates that intratumoral desmoplastic stroma (see Figure 1) is not a universal feature of BMs but a lesion-specific phenomenon detectable in at least every fourth carcinoma BM. It occurs significantly more in certain BMs, e.g., breast cancer metastases, and cannot be easily explained by clinicopathological variables such as age, sex, anatomical location, PD-L1 expression, or common driver alterations [7].

Recent single-cell and spatial transcriptomic studies provide stronger evidence that at least a subset of BMs contains bona fide fibroblast-like or CAF-like populations with extracellular matrix programs distinct from glial and endothelial compartments.

This complexity is particularly relevant when considering differences between tumor entities and growth patterns. Melanoma metastases, for example, often grow by vascular co-option and may exploit pre-existing host vasculature rather than establishing a classical collagen-rich intratumoral stroma. By contrast, carcinoma metastases more often appear capable of generating collagen-rich stromal microenvironments, although such features remain heterogeneous in frequency and extent even within this group.

Before turning to their origin, it is worth recalling why CAF-like cells matter in the first place. In tumors throughout the body, they reshape the extracellular matrix, provide signals that help tumor cells survive and invade, promote new blood vessels, and foster an environment that weakens the antitumor immune response, and they are increasingly linked to resistance against chemotherapy, radiotherapy, and immunotherapy [8]. CAFs are also not a single uniform cell type but comprise several functionally distinct subpopulations that may coexist within the same tumor [9].

If the CAF-like cells in brain metastases arise from different cellular origins, they probably carry different markers and depend on different signals, which could allow future stratification by lineage or marker profile, perhaps comparable to the way receptor status guides treatment in breast cancer, and ultimately more selective, stroma-directed therapy.

Against this background, the central question is no longer merely whether CAF-like cells exist in BMs but from which cellular sources they arise and by which biological routes they are generated.

In this review, we therefore discuss seven non-mutually exclusive hypotheses for the origin of intratumoral CAF-like cells in BM (see Graphical Abstract and Table 1 for a structured comparison). We propose these hypotheses as a conceptual framework to organize the current evidence and do not intend them to be exhaustive. By comparing them systematically, we aim to identify the best-supported mechanisms, the major evidentiary gaps, and the implications for a biologically meaningful classification of stromal cell states in BM.

## 2. Hypothesis 1—Pseudostroma

BMs frequently grow along pre-existing vascular structures and may thereby entrap normal or reactively altered brain parenchyma between tumor cell clusters. In this regard, different carcinoma types display distinct growth patterns. In particular, infiltrative growth patterns may give rise to areas that histologically resemble stroma but in fact consist of residual or reactively transformed CNS components.

Reactive astrocytes not only form a peritumoral response zone but may also infiltrate deeply into metastatic lesions, where they can establish a dense fibrillary network that may easily be mistaken for fibroblastic stroma in conventional histological preparations. Single-cell analyses have shown that, in addition to tumor and immune cells, BMs also contain oligodendrocytes and neural stem cell-like populations [10], at least partly supporting the presence of entrapped brain parenchyma.

At the same time, however, scRNA-seq, spatial transcriptomic, and protein-based studies have consistently identified cell populations in BM with clearly fibroblastic or CAF-like profiles [10,35,36]. These cells express canonical mesenchymal markers such as COL1A1, COL1A2, COL3A1, DCN, and FAP and are transcriptionally distinct from astrocytes, pericytes, and endothelial cells [35].

Furthermore, whereas remodeled brain parenchyma would be expected to be characterized primarily by brain-specific matrix components such as hyaluronic acid, proteoglycans, and laminin, fibrotic BMs exhibit deposition of collagen types I and III, fibronectin, and other constituents of a genuine desmoplastic stroma.

This may help explain why BMs display markedly increased tissue stiffness at the biophysical level [37]. While studies have shown that glial scars [38] and melanoma metastases [39] are mechanically softer than healthy brain tissue, this does not appear to apply to carcinoma metastases. At the same time, melanoma metastases, unlike carcinoma metastases, do not exhibit a typical CAF-like stroma, but instead usually show densely packed tumor cells morphologically [7] and induce pronounced astrogliosis at an early stage, which promotes tumor growth [11]. Notably, this stroma-forming capacity was not confined to a single case (0/75) in our own cohort but was observed across all carcinoma types we examined, including breast, pulmonary, colorectal, upper-gastrointestinal, prostatic, renal, and urothelial carcinomas, whereas it was consistently absent in melanoma [7].

Taken together, the differences in tissue stiffness between melanomas and carcinomas are therefore more readily explained by desmoplasia and collagen deposition than by gliosis alone or by tumor cell density. Accordingly, a purely pseudostromal component appears more plausible in melanoma metastases, whereas carcinoma metastases may contain both components.

In early stages of colonization, in infiltrative marginal zones, or in lesions dominated by vessel co-option [40], remodeled parenchyma consisting of reactive astrocytes, neuropil, and other resident CNS cells is likely to contribute to the stroma-like microenvironment. In more fibrotic and biologically advanced regions, however, genuine fibroblastic stromal compartments with a CAF phenotype, collagen-rich matrix, and functionally tumor-promoting activity become increasingly prominent. Thus, the original question remains: where do the CAF cells come from?

## 3. Hypothesis 2—Meningeal/Pial Cells

The meninges harbor precisely those cell populations that would, in principle, be capable of establishing a collagen-rich stromal compartment within the brain. Embryological studies indicate that the anterior meninges arise from the neural crest, whereas the posterior meninges originate from the mesoderm, and that fibroblasts of the individual meningeal layers—pial, arachnoidal, and dural—display distinct expression signatures. Pial fibroblasts in particular express ECM-related genes, including collagens, laminins, matrix metalloproteinases, and proteoglycans [12,41].

Notably, the pia mater accompanies penetrating blood vessels deep into the parenchyma. Pial cells and perivascular fibroblasts (PVFs) are transcriptionally very similar, if not identical [42]. Lineage-tracing studies have shown that PVFs in the adult brain do indeed derive from meningeal precursor cells and migrate along the vascular tree during postnatal development [13]. Under pathological conditions such as stroke or traumatic injury, these cells leave their perivascular niche, invade the parenchyma, and form fibrotic scars [43].

Against this background, it appears mechanistically plausible that metastatic tumor cells recruit fibroblastic cells through wound-healing and inflammatory signals such as PDGF, IL-1, or matrix metalloproteinases (MMP9) and drive them into an activated, matrix-producing state [44,45,46].

Recent studies have shown that medulloblastoma metastases in the leptomeninges are able to recruit meningeal fibroblasts via PDGF signaling. These fibroblasts are then reprogrammed into metastasis-associated meningeal fibroblasts (MB-MAFs), which in turn produce signals, most notably BMP4/7, that support tumor cell engraftment and further growth [14]. Although medulloblastomas are embryonal CNS tumors arising in the posterior fossa, these studies demonstrate that meningeal fibroblasts can be reprogrammed into MAFs and thus, in principle, also into CAFs.

However, several substantial arguments speak against a meningeal origin. Because BMs reach the brain via hematogenous spread, they tend to localize at the gray–white matter junction and in vascular border zones, where the diameter of arterioles narrows [47]. They are therefore usually situated deeper within the parenchyma and at a distance from the actual meninges. This would be more readily explained by PVFs, which closely resemble the pial meninges.

Classical ECM markers such as collagens, decorin, lumican, FAP, PDGFRβ, or α-SMA lack sufficient specificity, as they may be expressed not only by meningeal cells and perivascular fibroblasts but also by other vascular cells and, in part, by mesenchymalized tumor cells. A meningeal fibroblast origin could only be inferred if layer-specific meningeal markers were additionally demonstrated; however, this is not evident from the literature thus far, which is why the observed profile often appears more consistent with perivascular fibroblasts [10,35].

In RNA-seq-based approaches, spatial information is largely lost, so it often remains unclear whether a fibroblastic cluster is truly intratumoral or instead derives from the meninges, the vascular adventitia, or adjacent tissue. In addition, particularly in superficially sampled specimens, contamination with dural or pial components is possible, which may artificially reinforce the impression of a meningeal stroma.

## 4. Hypothesis 3—Vascular Cells

According to this hypothesis, CAFs arise from vascular (endothelial, vascular smooth muscle) or perivascular cell populations, with an endothelial origin being supported by the least evidence. All of these cells contribute to the maintenance of the blood–brain barrier. However, within the metastatic milieu, tumor-derived signals may induce them to adopt activated, stroma-forming states.

Mechanistically, three processes may be considered: the expansion of pre-existing perivascular fibroblastic cells (PVFs; see Hypothesis 2), the pericyte-to-fibroblast transition, and an endothelial-to-mesenchymal transition.

An analysis of 40 BMs suggested that pericytes are the most likely source of fibrotic stroma, based on the presence of PDGFRβ- and HSP47-positive perivascular cells whose distribution closely correlated with collagen deposition. This was further supported by the observation that the outer pericyte layer may detach from vessels and reappear as collagen-associated cells at the tumor margin or within the tumor itself [15].

This may be explained by the fact that metastatic cells use pre-existing vessels as guiding structures, thereby coming into direct contact with the vascular wall and potentially promoting mural cells to undergo activation or redirection into stroma-forming programs [48].

Accordingly, one study showed that tumor cells secrete PDGF-BB, which binds to PDGFRβ on pericytes, allowing them to detach from vessels and lose their typical pericyte marker NG2. Through this pathway, they may then convert into CAFs and acquire markers such as FSP1 and PDGFRα [16].

More recent single-cell and transcriptomic studies in BM support this hypothesis by identifying vascular cells (endothelial and mural) with partly immunoregulatory and matrix-producing properties. Beyond ECM-associated pericyte and fibroblast populations, BMs showed increased collagen IV deposition and marked vascular permeability. Endothelial–mural communication was also particularly prominent and involved collagen, laminin, fibronectin, and PDGF signaling, supporting a role for vascular cells in matrix remodeling and metastatic niche formation [17,18].

Further support comes from findings in human samples demonstrating PDGFRβ- or αSMA-positive stromal cells, as well as from experimental data indicating that pericytes can promote the growth of BM through secreted factors such as IGF2 [19,49]. In breast cancer BM, PDGFRβ-expressing CAFs release particularly high levels of fucosylated proteins, most notably PVR/CD155, which on the tumor cell side primarily alters cell–cell contacts and the actin cytoskeleton, and in mouse models also leads to increased invasiveness [50].

Perivascular fibroblasts or pericyte-like cells have also been increasingly identified in gliomas, particularly glioblastomas, where they are progressively being associated with resistance to immunotherapies and chemotherapies [51,52,53,54]. These findings would indirectly support the notion that CAFs in BM may also derive from cell populations that are genuinely resident within the brain parenchyma.

## 5. Hypothesis 4—Glial Cells

In non-neoplastic settings of CNS injury, reactive astrocytes have been shown to synthesize extracellular matrix components, express collagens, and contribute to both glial and fibrotic scar formation [20,55].

BMs are capable of inducing and maintaining a pSTAT3-positive subpopulation of reactive astrocytes that is functionally distinct from pSTAT3-negative astrocytes. Functionally, these cells promote the growth of BM by attenuating local CD8 T-cell responses and influencing CD74-positive microglia/macrophages [56].

Furthermore, they are preferentially localized at the brain–tumor interface, where they support invasion into the brain parenchyma through STAT3-dependent factors such as CHI3L1 [57]. High pSTAT3 expression in reactive astrocytes in triple-negative breast cancer BM is associated with higher intracranial recurrence rates and shorter progression-free survival [58].

A study on glioblastomas supports the hypothesis that reactive astrocytes may undergo reprogramming. It showed that activation of mTOR in astrocytes induces the synthesis of collagens such as COL5A1 and chaperones such as SERPINH1, thereby marking a transition toward a CAF-like state [21]. However, these studies do not demonstrate a direct glial-to-mesenchymal transformation, but rather a CAF-like activation or remodeling of astrocytes.

Another potential player is NG2 glia, usually understood as oligodendrocyte precursor cells (OPCs), which represent a highly plastic glial cell population of the CNS. They appear interesting as a potential source of CAF-like cells because, following tissue injury, they rapidly proliferate, migrate, participate in repair processes, and may undergo transient state changes under pathological conditions [22,59].

However, the current literature also only supports an indirect role of these cells in the modulation of the vascular niche, the blood–brain barrier, angiogenesis, and possibly early tumor colonization rather than a directly demonstrated differentiation into classical fibroblastic stromal cells [60].

Moreover, in many datasets, astrocytic clusters and fibroblastic clusters appear clearly separated, without obvious transitional states [10,18]. Typical CAF markers such as FAP, PDPN, or collagens are usually not found together with classical astrocytic markers such as GFAP, AQP4, or SLC1A2 [61].

Overall, glial cells may well assume a matrix-producing, scar-like, and functionally CAF-related role; however, this most likely reflects functional convergence rather than a genuine glial-to-mesenchymal transformation into CAFs.

## 6. Hypothesis 5—Cancer Stem Cells

Carcinoma cells can, in principle, switch between epithelial, mesenchymal, stem-like, neural-like, and other adaptive programs [62]. Recent studies suggest that the relevance of epithelial–mesenchymal transition (EMT) to metastasis lies not in fully mesenchymal end states but rather in plastic intermediate or hybrid EMT states.

Using scRNA-seq datasets from several tumor types, combined with mathematical modeling, it was shown that EMT represents a continuum with stable intermediate states and that these intermediate states are characterized by shared markers such as SFN and NRG1 [63].

In a mouse model of metastatic pancreatic carcinoma, inducible CRISPR–Cas9 lineage tracing showed that metastatic potential is greatest in rare late hybrid EMT states derived from a predominantly epithelial founder pool. The study also demonstrated a marked clonal bottleneck, as only a few subclones successfully formed metastases [23].

CD44+ cancer stem cells (CSCs) can adopt a pericyte-like state that promotes intravasation, survival in circulation, brain extravasation, and subsequent reversion to tumorigenic CSCs. This process depends on GPR124/Wnt7–β-catenin signaling, and disruption of this axis markedly reduces BM formation [24]. In the brain microenvironment, CSCs may further upregulate CD146, thereby strengthening pericyte-like features and promoting angiogenesis via enhanced VEGF/VEGFR2 signaling, a state further reinforced by reactive astrocytes through GAS6/AXL signaling [25].

Studies in mouse models showed that invasive cells in breast cancer BM activate an EMT-associated mesenchymal program at the tumor front, detach from the tumor cell collective, and penetrate further into the brain parenchyma. At the same time, these cells remain plastic and may later reacquire more epithelial states. This hybrid phenotype was also supported in human tissue, where 7 of 8 patient samples contained ZEB1-positive, E-cadherin-negative, yet pancytokeratin-positive tumor cells within the brain parenchyma [26].

A separate analysis of tumor and stromal transcriptomes in xenograft BM further confirmed that metastatic carcinoma cells in the brain display pronounced plasticity, activating EMT, WNT, motility, and neural-like programs, while the surrounding brain stroma develops a marked neuroinflammatory response with reactive astrocytes, microglia, and TAMs. In this setting as well, the tumor cells were able to co-express CDH1 together with ZEB1 and VIM, supporting a partial or hybrid EMT/MET state [1].

Carcinoma cells may therefore adopt hybrid states in which the tumor cell activates stroma-like programs without losing its epithelial identity or morphology. This plasticity may explain seemingly contradictory sequencing datasets in which mesenchymal or hybrid EMT programs appear more prominent than would be expected from histological assessment. However, direct evidence that CSCs can differentiate into a permanent intratumoral stromal component is still lacking.

## 7. Hypothesis 6—Tumor–Stroma Clusters

CAF-like cells in BMs need not arise exclusively through local reprogramming within the CNS but may instead derive from tumor–stroma clusters that are formed already before or during hematogenous dissemination.

This model is biologically plausible, first of all, in light of the well-established organization of collective migration within the primary tumor. In addition, CAFs at the invasive front are able to actively organize migration through direct cell–cell contacts, TGF-β-mediated signaling, and ECM remodeling [27,28].

Thus, the primary tumor already provides the conditions for the formation of heterotypic assemblies in which tumor cells and accompanying fibroblastic cells jointly exploit invasive tracks and may potentially enter blood vessels together [29].

Heterotypic clusters of circulating tumor cells (CTCs) possess additional functional advantages over single cells. Recent studies suggest that clusters containing platelets and neutrophils enhance tumor cell survival through paracrine and contact-dependent support from accompanying cells [64,65].

Co-disseminated CAFs may mechanically stabilize the cluster through direct cell adhesion contacts. This is supported by the detection of heterotypic CAF-CTC clusters in patients and in mouse models, as well as by the involvement of CD44 in their cluster formation. Moreover, such heterotypic clusters display greater metastatic potency in vivo than homotypic CTC clusters [30].

The most critical step is the passage into the brain parenchyma. Direct proof that intact heterotypic tumor–CAF clusters can traverse the blood–brain barrier and, as such, find the initial lesion is lacking. Nevertheless, several mechanistic arguments render this scenario plausible.

First, the size of collective tumor units favors their arrest in cerebral capillaries and vascular branch points, thereby prolonging their interaction with the endothelium. Preclinical models show that tumor cells arrested already within the vasculature can trigger local plasmatic coagulation with VWF deposition and microthrombus formation, which further stabilizes vascular arrest and promotes both extravasation and subsequent macrometastatic outgrowth [66,67].

Second, accompanying stromal and vascular components, including coagulation factors, VWF-rich microthrombi, and leukocytes, may promote adhesion and vascular permeability. Tumor cell-induced vascular occlusion may further favor early brain colonization by disrupting cerebral microcirculation and inducing a hypoxic–ischemic microenvironment [68].

Third, active endothelial remodeling and angiopellosis-like processes have been described. Angiopellosis differs from classical diapedesis in that the circulating cell is not the primary active element; rather, the vascular endothelium itself organizes the exit, thereby allowing larger cellular aggregates to be expelled into the tissue [69,70].

Once tumor cells reach the brain parenchyma, they find themselves in an environment that is hostile to epithelial carcinoma cells. Co-disseminated fibroblastic cells could deposit extracellular matrix at an early stage, provide integrin-mediated survival signals, and release soluble mediators, thereby generating a “portable niche”.

## 8. Hypothesis 7—Circulating Stem Cells

Several carcinoma models have shown that bone marrow-derived mesenchymal stromal cells (BM-MSCs), recruited via the circulation, can migrate into the tumor microenvironment and differentiate there into CAFs or CAF-like cells.

In a model of inflammation-induced gastric carcinogenesis, it was demonstrated that at least 20% of CAFs originate from the bone marrow and are derived from MSCs; these cells were also functionally linked to tumor progression [31]. Similarly, in mouse models of H. pylori-associated gastric carcinoma, GFP-labeled BM-MSCs migrated into gastric tissue and differentiated into α-SMA-positive CAFs. In addition, donor-derived cells were identified in human gastric tissue following allogeneic bone marrow transplantation [71].

In an experimental model of triple-negative breast carcinoma, BM-MSCs were shown to promote the aggressive behavior of tumor cells, and blockade of PDGFRβ inhibited both their homing to the tumor and their transdifferentiation into CAF-like cells [32].

Similarly, in a transgenic breast carcinoma model with spontaneous lung metastases, BM-derived MSCs were shown to contribute substantially to CAF formation in the primary tumor and to be recruited also to lung metastases. The authors further demonstrated that the relative decline of PDGFRα-positive CAFs during tumor progression was not attributable to a loss of resident fibroblasts, but rather to the recruitment of a second, PDGFRα-negative CAF subpopulation from the bone marrow. Using bone marrow transplantation, genetic fibroblast reporters, and functional analyses, they showed that these cells arise from BM-derived MSCs, differentiate into Col1α-positive CAFs only within the tumor microenvironment, and differ from resident CAFs both phenotypically and functionally [33].

Evidence from prostate carcinoma likewise showed that TGF-β1 derived from both tumor and stroma promotes MSC recruitment and drives their conversion into CAF-like cells [72].

To date, however, no direct lineage-tracing evidence has been provided for an origin of CAFs in BM from BM-MSCs. Nevertheless, chimeric mouse models with GFP-labeled bone marrow have shown that bone marrow-derived CD11b+ myeloid cells, or HSC-derived myeloid progeny, are capable of infiltrating BM, indicating that the blood–brain barrier does not in principle preclude the recruitment of bone marrow-derived cells [34].

## 9. Integrative Discussion

Taken together, the available evidence suggests that CAF-like cells in BM are unlikely to arise through a single universal mechanism. Rather, the current literature supports a heterogeneous and context-dependent model in which different routes of stromal emergence may predominate depending on tumor entity, molecular subtype, anatomical location, growth pattern, and stage of metastatic colonization.

In this framework, the concept of a purely reactive pseudostroma remains important, particularly for early and infiltrative lesions, but it does not explain the full stromal spectrum observed in BMs. While parts of the stromal-appearing compartment may indeed reflect entrapped or reactively remodeled brain tissue, single-cell and spatial analyses repeatedly identify distinct fibroblast-like populations with collagen-rich extracellular matrix programs.

Among the proposed origins, vascular and perivascular lineages currently appear the most plausible. BMs develop in intimate spatial and functional association with the cerebral vasculature, and the perivascular niche represents one of the earliest and most active sites of metastatic adaptation. This makes pericytes and perivascular fibroblast-like cells particularly compelling candidates, as these populations are already equipped with matrix-associated programs under physiological conditions and appear capable of transitioning into more overtly fibrogenic states in tumor settings.

The convergence of histological observations, experimental data on pericyte-to-fibroblast transition, and more recent single-cell atlases of endothelial and mural compartments provides the strongest support for this hypothesis.

At the same time, the meningeal hypothesis should not be regarded as mutually exclusive. Rather, it may be incorporated into a broader developmental model, since perivascular fibroblasts of the brain can themselves derive from meningeal precursor populations. Accordingly, a more direct meningeal contribution may be conceivable in superficial or leptomeningeal lesions, whereas in deeply parenchymal metastases, a perivascular, meningeal-derived fibroblast-like origin appears biologically more plausible than direct recruitment of classical meningeal cells into the tumor core.

By contrast, the glial, cancer stem cell, and bone marrow-derived precursor hypotheses remain biologically relevant but currently less well supported as dominant explanations for bona fide CAF-like populations.

Astrocytes clearly play a major role in BMs by shaping invasion, neuroinflammation, and metastatic outgrowth, and some studies suggest that they can adopt matrix-associated and CAF-like activation states. However, the available data still argue more strongly for functional convergence than for a true lineage conversion into stable fibroblast-like stromal cells.

A similar caution applies to cancer stem cell-related models. Hybrid EMT states and pericyte-like tumor cell programs convincingly illustrate how metastatic tumor cells can transiently acquire stromal traits and exploit them for dissemination and colonization, yet direct evidence that such states generate a durable fibroblast-like intratumoral compartment is still lacking.

Likewise, the notion that bone marrow-derived mesenchymal precursors may contribute to CAF-like cells in BM remains plausible, particularly because bone marrow-derived cells can infiltrate metastatic lesions, but direct lineage-based proof for this route in human BMs is still absent.

A central conclusion, therefore, is that no two BMs should be expected to display identical stromal biology. Each lesion represents its own biological situation, shaped by the interplay between tumor-intrinsic programs and the specific microenvironment encountered in the brain. Some metastases may be dominated by myeloid-rich and glia-reactive niches in which classical fibroblast-like stromal elements remain limited, whereas others may develop a more distinct fibrotic, collagen-rich, and perivascularly organized CAF-like compartment.

Importantly, these patterns are not necessarily mutually exclusive. Mixed phenotypes are likely and may even coexist within a single lesion, for example, when reactive glial and vascular remodeling predominates at the invasive margin while a more established fibroblast-associated matrix niche emerges in the lesion center. The most realistic model is therefore not an either/or scenario but a dynamic spectrum of overlapping stromal states that may vary across patients, across metastases, and even across spatial regions of the same metastasis.

This heterogeneity is not only conceptually important but may also have therapeutic implications. At present, however, functional models capable of distinguishing the specific roles of individual CAF-like populations in BM are still limited. Consequently, grouping all CAF-like cells under a single umbrella term risks obscuring biologically distinct cell states. While these populations may share selected markers, they are unlikely to make equivalent contributions to invasion, immune modulation, treatment resistance, or metastatic persistence.

## 10. Conclusions

The origin of CAF-like cells in brain metastases cannot be reduced to a single mechanism. The current literature points instead to several parallel routes, and which of them dominates probably depends on the primary tumor, its molecular subtype, and the location, growth pattern, and stage of the metastasis. Of the seven hypotheses discussed here, a vascular and perivascular origin is at present the best supported, with pericytes and perivascular fibroblast-like cells as the most convincing candidates. A glial, cancer-cell, or bone-marrow origin remains conceivable but rests on weaker evidence, and more than one route may operate within the same lesion. The question is also of practical importance, since CAF-like cells of different origin do not need to behave alike in invasion, immune escape, or response to treatment, and a clearer view of where they come from is a precondition for defining reliable markers and for developing stroma-directed therapies. So far, direct lineage-tracing data in human brain metastases have been limited, and closing this gap will be the decisive next step.

## Figures and Tables

**Table 1:** Overview of the seven main hypotheses on the origin of CAF-like cells in brain metastases, including their conceptual basis, the spatial contexts in which they may manifest as stromal elements, the methodological approaches supporting them, their key limitations, and their overall plausibility. Abbreviations: BM, brain metastasis; CAF, cancer-associated fibroblast; ECM, extracellular matrix; EMT, epithelial–mesenchymal transition; OPC, oligodendrocyte precursor cell; BBB, blood–brain barrier.

Hypothesis	Statement	Spatial Context	Key Reference	Key Limitation	Plausibility (Authors’ Opinion)
1. Pseudostroma	Stroma (partly) represents entrapped or reactive brain parenchyma	Invasive margin, melanomas	[2,6,10,11]	Does not explain collagen I/III-rich niches or distinct fibroblast/CAF-like clusters	Moderate (stroma-like appearance); low (true stroma)
2. Meningeal/pial cells	Meningeal-derived cells are recruited or activated into CAF-like cells	Superficial/leptomeningeal	[12,13,14]	Hard to prove in deep parenchymal metastases; profiles may fit better to perivascular fibroblasts	Moderate
3. Vascular cells	Pericytes or perivascular cells transform into matrix-producing CAF-like cells	Intratumoral, but no special location	[15,16,17,18,19]	Endothelial origin weakly supported, perivascular origin well supported, but definitive BM-specific lineage tracing remains limited	Moderate–High
4. Glial cells	Reactive astrocytes or NG2-glia adopt CAF-like, matrix-remodeling functions	Invasive margin	[20,21,22]	Functional convergence rather than true glial-to-fibroblast conversion	Low
5. Cancer stem cells	Tumor cells in hybrid EMT- or pericyte-like states mimic CAF-like behavior	Intratumoral, but no special location	[23,24,25,26]	Strong for plasticity and stromal mimicry, but not for stable non-neoplastic CAF compartment	Moderate
6. Tumor–stroma clusters	CAF-like cells co-disseminate with tumor cells as heterotypic clusters	Intratumoral, but no special location	[27,28,29,30]	No direct proof that intact tumor-CAF clusters cross the BBB to seed BM in vivo	Low–Moderate
7. Circulating stem cells	Bone marrow-derived MSCs home to BM and differentiate into CAF-like cells	Intratumoral, but no special location	[31,32,33,34]	Plausible, but no direct lineage-based proof for this route in BM	Low–Moderate

## Data Availability

No new data were created or analyzed in this study. Data sharing is not applicable to this article.
